# Editorial: Control of plant organ size and shape

**DOI:** 10.3389/fpls.2022.1067587

**Published:** 2022-10-26

**Authors:** Ran Xu, Shengjun Li, Na Li, Hirokazu Tsukaya, Yunhai Li

**Affiliations:** ^1^ Sanya Nanfan Research Institute of Hainan University, Hainan Yazhou Bay Seed Laboratory, Sanya, China; ^2^ College of Tropical Crops, Hainan University, Haikou, China; ^3^ Key Laboratory of Biofuels, Shandong Provincial Key Laboratory of Energy Genetics, Shandong Energy Institute, Qingdao Institute of Bioenergy and Bioprocess Technology, Chinese Academy of Sciences, Qingdao, China; ^4^ State Key Laboratory of North China Crop Improvement and Regulation, Key Laboratory of Vegetable Germplasm Innovation and Utilization of Hebei, Collaborative Innovation Center of Vegetable Industry in Hebei, College of Horticulture, Hebei Agricultural University, Baoding, China; ^5^ Graduate School of Science, The University of Tokyo, Tokyo, Japan; ^6^ State Key Laboratory of Plant Cell and Chromosome Engineering, Chinese Academy of Sciences (CAS) Center for Excellence in Molecular Plant Sciences, Institute of Genetics and Developmental Biology, Chinese Academy of Sciences, Beijing, China

**Keywords:** plant, organ size, organ shape, leaf, floral organ, seed, fruit

Organ size and shape are the most intuitive morphological characteristics of plants. Dissecting the mechanisms controlling plant organ size and shape could shed light on the nature of plant diversity. In addition, organ size and shape directly associate with crop yield and quality. Identification of key regulators of organ size and shape would provide new genetic targets for crop improvement. This Research Topic aims to illustrate the latest advances of plant organ size and shape regulation.

In this Research Topic, two review articles summarized morphology and organ size control in soybean and the role of salicylic acid (SA) in plant growth, respectively. During the domestication process of soybean, several significant morphology and organ size changes have occurred, such as twisted stems to erect stems and small seeds to large seeds. Zhou et al. summarized genes participated in stem growth habit, leaf size and shape, and seed size and weight in soybean. They also discussed the application of new technologies on the basic research and breeding of soybean and the challenges and hotspots of soybean research in the future. Salicylic acid (SA) is an important phytohormone that not only plays critical roles in plant immunity, but also is involved in plant growth regulation. Li et al. summarized current knowledge about the roles of SA on plant growth. The authors highlighted that SA mediates growth regulation by affecting both cell division and expansion. Particularly, they discussed the interactions of SA with other hormones in plant growth determination. Fiber length is one of the most important economic traits of cotton. In this Research Topic, Pandey et al. provided an opinion article that presents novel perspectives about evolution of fiber length. They expounded that concurrent transcriptional dynamics of cytoskeleton-associated structural genes in modern cotton fibers are very useful in understanding the evolutionary recruitment of cell wall-modifying gene clusters for shaping the floral organs and determining fiber length.

Identification of novel regulators of organ size and shape is of great importance in understanding the molecular mechanisms underlying plant growth and development control. In this Research Topic, several novel factors involved in organ size and shape control were characterized. Gao et al. described the role of BrCPS1 (ENT-COPALYL DIPHOSPHATE SYNTHASE 1) in leafy head formation, which is an important agronomic trait in Chinese cabbage (*Brassica rapa* L. ssp. pekinensis). *BrCPS1* affects gibberellin biosynthesis, and mutants of *BrCPS1* did not form leafy heads at the heading stage, revealing the critical role of BrCPS1-mediated gibberellin biosynthesis in leafy head formation. FW2.2 is a classical regulator of fruit size in tomato (Frary et al., 2000). Wang et al. showed that the homolog of FW2.2, MdCNR8 (Cell Number Regulator 8), is sumoylated by the SUMO E3 ligase MdSIZ1 to control organ size in apple, suggesting that FW2.2/CNR8 have a conserved function in fruit size control in tomato and apple. TTG1 participated in trichome formation and anthocyanin accumulation in *Arabidopsis* (Zhang et al., 2003; Chen et al., 2015). Huang et al. showed that RrTTG1 may promote fruit prickle development through an MBW complex in *Rosa roxburghii*, which revealed the conserved roles of TTG1 in different species and laid a ground for genetic improvement of prickle-free *R. roxburghii*. Zhao et al. demonstrated that ectopic expression of poplar *PsnCYCD1;1* in *Nicotiana tabacum* regulated flower organ development, which provides new understanding for CYCD function. Besides for these new regulators of organ size and shape, Xie et al. identified a specific mutant (referred as *mr1*) with a reduced palea in rice, and mapped the causal gene of *mr1*, which will contribute to understanding of grain formation. Liu et al. identified QTLs associated with agronomic traits in tobacco *via* a biparental population and an eight-way MAGIC population, which could be applied for breeding new tobacco varieties using molecular marker-assisted selection. Based on a high-quality genetic map constructed by whole genome resequencing, An et al. identified 25 QTLs affecting leaf area of tea plants and developed the related molecular markers, providing useful tools for molecular breeding of tea plant.

Flowering time could affect the final morphology and biomass of plants. It is known that vernalization induces epigenetic silencing of the floral repressor gene *FLC* (*FLOWERING LOCUS C*) (Michaels and Amasino, 1999; Whittaker and Dean, 2017; Zhu et al., 2021). In this Research Topic, Maruoka et al. found that the histone demethylases JMJ30 and JMJ32 could brake vernalization through the activation of *FLC*, which provides novel insight about the role of repressive histone marks in environmental responses in plants. FT (Flowering locus T) is a key flowering regulator (Turck et al., 2008). Wu et al. introduced an FT homolog from *Jatropha curcas* into tobacco, and found that FT played crucial role in stem growth.

In addition to the above articles, the other six articles provided some novel information about organ size and shape from other aspects. By performing systematic analyses of the cytological characteristics and underlying mechanism of morphological variation in culms of *Phyllostachys nidularia f. farcta* (Shidu bamboo), Wang et al. found that the decrease in the total number of internodes and the decrease in dry matter content may contribute to the sharp decline in culm biomass of Shidu bamboo. Langer et al. investigated twist-to-bend ratios and safety factors of petioles with various geometries, sizes and shapes, and provided an equation to calculate the safety factor of naturally horizontally oriented petioles for the first time. KNOX genes encode homeobox transcription factors that play critical roles in determining cell fate in shoot apical meristem (Meng et al., 2020). Zhang et al. performed genome-wide identification of KNOX gene family in Orchidaceae, and provided a comprehensive analysis to uncover the underlying function of KNOX genes in Orchidaceae. Tu et al. performed a comprehensive analysis of the role of long non-coding RNAs, microRNAs, and transcription factors in regulating leaf and flower development in *Liriodendron chinense*, which laid a foundation for further investigation into the regulatory mechanisms of leaf and flower development in *Liriodendron chinense*. By CRISPR/Cas9-mediated editing in the cis-regulatory element of *KLUH* promoter, the fruit weight of tomato was significantly increased (Li et al). By employing mutants with thicker lateral roots, Kawai et al. found that auxin distribution in lateral root primordium affects the size and lateral root diameter in rice. Altogether, this Research Topic provides the latest progresses on the regulation of plant organ size and shape by uncovering new molecular mechanisms and key regulators.

## Author contributions

All authors listed have made a substantial and direct contribution to the work and approved it for publication.

## Acknowledgments

We are grateful to all authors, journal editors, and peer reviewers who contributed to this Research Topic.

## Conflict of interest

The authors declare that the research was conducted in the absence of any commercial or financial relationships that could be construed as a potential conflict of interest.

## Publisher’s note

All claims expressed in this article are solely those of the authors and do not necessarily represent those of their affiliated organizations, or those of the publisher, the editors and the reviewers. Any product that may be evaluated in this article, or claim that may be made by its manufacturer, is not guaranteed or endorsed by the publisher.
